# Effects of Nicotinamide Mononucleotide Supplementation on Blood Pressure: A Systematic Review and Meta-Analysis of Randomized Controlled Trials

**DOI:** 10.3390/nu18060890

**Published:** 2026-03-11

**Authors:** Mu Zhang, Yingci Chen, Nan Jiang, Jingjing Zeng, Jianyun Zhang, Chenyang Wu, Yingying Liu, Zizheng Nie, Jun Yang, Shufen Han

**Affiliations:** Department of Nutritional and Toxicological Science, School of Public Health and Nursing, Hangzhou Normal University, Hangzhou 311121, China; 2024112024010@stu.hznu.edu.cn (M.Z.); 2025112026003@stu.hznu.edu.cn (Y.C.); 2023211506089@stu.hznu.edu.cn (N.J.); 2023211506081@stu.hznu.edu.cn (J.Z.); jianyunz@hznu.edu.cn (J.Z.); 2024112024009@stu.hznu.edu.cn (C.W.); 2023112024022@stu.hznu.edu.cn (Y.L.); 2022111027004@stu.hznu.edu.cn (Z.N.)

**Keywords:** nicotinamide mononucleotide, supplementation, blood pressure, meta-analysis

## Abstract

**Background/Objectives:** High blood pressure remains a primary modifiable risk factor for cardiovascular disease. Nicotinamide mononucleotide (NMN) has emerged as a promising supplement; however, its efficacy with respect to blood pressure in humans is unclear. This meta-analysis systematically evaluated the effects of various NMN supplements on resting systolic blood pressure (SBP) and diastolic blood pressure (DBP) in adults with elevated blood pressure. **Methods:** A systematic literature search was conducted to identify eligible randomized controlled trials (RCTs) using the databases PubMed/MEDLINE, Scopus, Web of Science, and EBSCO from their inception to 13 December 2025. R software was used to combine the data from the included original trials using a common-effects model. Subgroup analyses were performed based on age, baseline body mass index, geographical location, intervention duration, NMN dosage, and baseline blood pressure. **Results:** A total of 349 participants from 10 RCTs with 11 intervention arms were included. Compared with the placebo, NMN supplementation was associated with a statistically significant but modest reduction in resting DBP (WMD, −2.15 mmHg; 95% CI: −3.68 to −0.61). In contrast, the reduction in resting SBP was not statistically significant. Notably, subgroup analyses revealed that NMN supplementation resulted in a significant reduction in SBP specifically among participants aged 60 years and older (WMD: −3.94 mmHg; 95% CI: −7.06 to −0.82). **Conclusions:** Our findings provide preliminary and suggestive evidence that NMN supplementation may be associated with a small reduction in resting DBP and a modest beneficial effect on resting SBP in adults aged 60 years and older. However, the potential of NMN as a viable candidate for early-stage blood pressure management requires confirmation through long-term, large-scale, high-quality RCTs in future clinical studies.

## 1. Introduction

The rapid growth of the aging global population has intensified the search for interventions to promote healthy aging. Among these, nicotinamide mononucleotide (NMN) has garnered considerable attention. As a bioactive nucleotide, NMN is naturally synthesized from a phosphate group and nicotinamide riboside through the enzyme nicotinamide phosphoribosyltransferase (NAMPT, EC 2.4.2.12) [1,2]. NMN acts as a direct precursor for the biosynthesis of nicotinamide adenine dinucleotide (NAD^+^), an essential cofactor in all living cells that is involved in fundamental biological processes including energy metabolism and cellular repair [3]. NMN has demonstrated promise in promoting healthy and productive aging [4]. The natural decline in NAD^+^ bioavailability with aging is well-documented [5,6,7,8]. Although NAD^+^ levels can be maintained through dietary intake of its classical precursor, niacin [9], recent research has increasingly focused on more direct precursors, such as NMN [10]. Given that the concentration of NMN in natural food sources is relatively low, there has been growing interest in direct NMN supplementation [11]. Although ongoing debates in various countries regarding whether NMN should be classified as a dietary supplement or a pharmaceutical treatment agent, an increasing number of clinical studies have positioned NMN supplementation as a viable option for health benefits [10,11,12], regardless of whether it is prescribed or self-administered. Preclinical studies suggest that boosting NAD^+^ concentrations through NMN supplementation can improve energy metabolism, reduce oxidative stress, mitigate DNA damage and neurodegeneration, and alleviate inflammatory responses [13,14,15,16,17]. Furthermore, existing clinical trials have demonstrated that oral NMN administration is generally safe and well-tolerated in humans within a defined dosage range [4,18], paving the way for investigating its potential therapeutic benefits.

Elevated blood pressure and hypertension represent a pervasive and consequential public health challenge, particularly among the aging global population. It is estimated that approximately 1.4 billion individuals aged 30 to 79 globally have hypertension in 2024, with an adequate control rate of less than 20% [19]. Elevated blood pressure is a leading preventable contributor to cardiovascular disease (CVD) mortality and disease burden in most regions around the world [20]. According to the guidelines from the American Heart Association and the American College of Cardiology [21], the new categories for blood pressure define “elevated blood pressure” as having a systolic blood pressure (SBP) ranging from 120 to 129 mmHg with a diastolic blood pressure (DBP) of less than 80 mmHg, while “stage 1 hypertension” is defined as a SBP between 130 and 139 mmHg or a DBP between 80 and 89 mmHg, and “stage 2 hypertension” is characterized by a SBP ≥ 140 mmHg or DBP ≥ 90 mmHg. The current guidelines emphasize stringent treatment goals to maintain blood pressure levels below 130/80 mmHg for all adults [21]. Although pharmaceutical treatments are effective for patients with hypertension, there is a pressing and growing need for non-pharmaceutical approaches, particularly for individuals in the pre-hypertensive or elevated blood pressure range. These non-pharmaceutical strategies, including nutrient-based and physical approaches, are designed to mitigate cardiovascular risk and prevent progression to overt hypertension [21].

The rationale for investigating NMN in blood pressure management is strongly supported by pathophysiology. A key mechanism underlying hypertension is endothelial dysfunction [22]. Promisingly, animal studies have shown that NMN supplementation, by boosting NAD^+^ levels, reduces oxidative stress and ameliorates endothelial dysfunction by restoring vascular Sirtuin 1 (SIRT1) activity [15,23]. In contrast, common nutrition items exert only generalized antioxidant effects without specifically modulating the NAD^+^ pathway [24]. This has led to the proposition that NAD^+^ boosting therapy may serve as a novel potential preventive or adjuvant strategy avenue for hypertension [25,26]. However, despite compelling mechanistic data and the availability of several human randomized controlled trials (RCTs), the clinical efficacy of NMN supplementation on blood pressure remains uncertain and inconsistent across studies. This critical gap between promising biological plausibility and unconfirmed clinical effectiveness necessitates a definitive synthesis of the existing evidence. Regarding other NAD^+^ precursors, such as nicotinamide riboside (NR), the existing research consensus indicates that NR supplementation alone is unlikely to exert a significant effect on blood pressure or arterial stiffness [25,27].

Consequently, the present study aimed to comprehensively evaluate the effects of oral NMN supplementation on blood pressure. To achieve this, we conducted a systematic review and meta-analysis of all peer-reviewed RCTs to quantify the effects of NMN supplementation on resting SBP and DBP in adults with elevated blood pressure, thereby providing evidence-based conclusions to inform both clinical practice and future research.

## 2. Methods

This systematic review was carried out in accordance with the Preferred Reporting Items for Systematic Reviews and Meta-analysis (PRISMA) guidelines [28]. The review protocol was registered in PROSPERO, the international prospective register of systematic reviews, prior to data extraction (registration number: CRD42025635763). Two authors (M.Z. and Y.C.) independently performed the literature search, study selection, data extraction, and quality assessment. Any discrepancies identified during the meta-analysis were resolved by consensus or through consultation with the senior authors.

### 2.1. Data Sources and Search Strategy

This meta-analysis was conducted and reported following the PRISMA guidelines (Table S1). A systematic literature search was conducted to identify pertinent studies published in English using four electronic databases, including PubMed/MEDLINE, Scopus, Web of Science, and EBSCO, from their respective dates of inception to 13 December 2025. The reference lists of the selected articles were also screened. Furthermore, a meticulous manual search of retrieved publications and relevant reviews was conducted to identify any additional eligible studies. The following search terms, including both MeSH and text words, were used to determine the query syntax: (“Nicotinamide mononucleotide” OR “Mononucleotide nicotinamide” OR “NMN” OR “NAD”) AND (“blood pressure” OR “BP” OR “hypertension”) AND (“clinical trials” OR “RCT” OR “intervention” OR “trial” OR “randomized” OR “crossover” OR “placebo”). The complete search strategy is presented in Table S2. It is noted that the corresponding authors were not contacted for additional information during the literature retrieval process.

### 2.2. Study Selection

The study selection process was guided by the PICOS framework (participants, interventions, comparisons, outcomes, and study design), detailed in Table S3. The inclusion criteria were as follows: (1) RCTs assessing the effects of NMN supplementation on blood pressure; (2) participants comprising adults aged 18 years or older who were healthy without other cardiovascular, diabetic or chronic underlying diseases; (3) a control group within a parallel or crossover design; (4) an intervention duration of four weeks or longer; and (5) provision of sufficient data to calculate the net changes in blood pressure from pre- to post-intervention. Studies were excluded if they were: (1) animal or in vitro studies; (2) non-randomized or non-primary studies, such as reviews, letters, conference abstracts, case reports, and observational studies (e.g., cross-sectional, cohort, or case–control studies); or (3) lacking sufficient information for pre- and post-intervention blood pressure. Where multiple publications reported on the same population, the most comprehensive or recent publication was selected.

### 2.3. Data Extraction

Two investigators (M.Z. and Y.C.) independently extracted relevant information from the included studies into a customized Microsoft Excel (version 16.0, Microsoft Corporation, Redmond, WA, USA) spreadsheet. The extracted information included the name of the first author, publication year, country of origin, study design details, sample size, participant characteristics (age, gender, baseline body mass index (BMI), and blood pressure), health status, intervention details (duration, NMN dosage and form), main results (pre- and post-intervention SBP and DBP), and reported adverse events. Extracted data were cross-verified, and disagreements were resolved through discussion among all authors.

### 2.4. Quality Assessment

The quality of the included RCTs was independently evaluated by two investigators (M.Z. and Y.C.) using the revised Cochrane Collaboration’s risk-of-bias tool 2.0 (RoB 2.0, The Cochrane Collaboration, London, UK) [29]. This tool evaluated the following five domains of bias: (1) bias arising from the randomization process, (2) bias due to deviations from the intended interventions, (3) bias due to missing outcome data, (4) bias in the measurement of the outcome, and (5) bias in selective reporting of outcomes. RevMan software (version 5.4, The Cochrane Collaboration, London, UK) was used to classify the risk of bias for each domain into three categories: “low risk of bias”, “some concerns” and “high risk of bias”.

### 2.5. Data Synthesis and Statistical Analysis

Blood pressure data were extracted as means and standard deviations (SDs). For data presented graphically, numerical values were extracted using WebPlotDigitizer (version 4.6; Ankit Rohatgi, San Francisco, CA, USA). If the SD was not provided in the publication, it was calculated from medians, standard errors, 95% confidence interval (CI), or interquartile ranges (IQR) using established standard formula from previous reports [30]. The intervention effect was evaluated by comparing the pre- and post-intervention differences in blood pressure between the NMN supplementation and placebo groups, and the net changes in resting SBP and DBP were calculated as the difference between the final and baseline blood pressure in each group, respectively. As blood pressure is a continuous variable, the overall effect size was expressed as a weighted mean difference (WMD) with a corresponding 95% CI.

Statistical analyses in the present study were performed using R software (version 4.4.3; R Development Core Team, Vienna, Austria). Heterogeneity of the effect size among trials was assessed using the Cochran’s Q test, with a significance level of *p* < 0.10. The degree of heterogeneity was quantified using the *I*^2^ statistics [31]. An *I*^2^ value exceeding 50% was considered to indicate substantial heterogeneity across trials. In the presence of significant heterogeneity, the random-effects model was adopted to assess the overall effect size. Conversely, when no substantial heterogeneity was detected (*I*^2^ < 50%), the common-effects model was used to calculate the overall effect size. To explore potential sources of heterogeneity across trials, we conducted pre-specified subgroup analyses based on the following factors: mean age, baseline BMI, study location, duration of supplementation, dosage of NMN, and baseline blood pressure. Sensitivity analyses were used to assess the impact of each individual study on the estimated overall risk and to examine sources of heterogeneity by sequentially excluding one study at a time. Any potential publication bias was assessed using Begg’s rank correlation test and Egger’s linear regression analysis [32]. A *p* value of less than 0.05 was considered statistically significant.

## 3. Results

### 3.1. Literature Research

**Figure 1:**
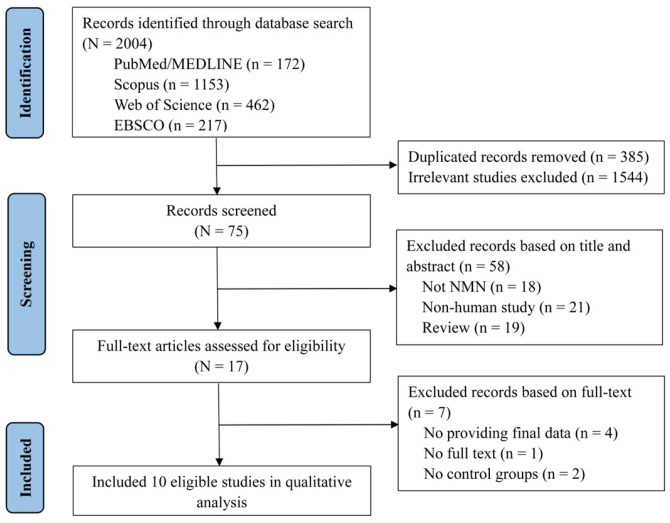
PRISMA flowchart of the literature search process and included studies.

A comprehensive literature search, from database inception to 13 December 2025, initially identified 2004 publications using the specified search terms. After excluding 385 duplicate documents and 1544 irrelevant studies based on their titles and abstracts, a total of 75 articles were retained for further review. Ultimately, 10 full-text articles were retrieved and included in the final analysis [26,33,34,35,36,37,38,39,40,41]. The PRISMA flowchart illustrating the study selection process is presented in Figure 1.

### 3.2. Study Characteristics

Table 1 summarizes the main characteristics of the 10 eligible RCTs with 11 intervention arms. One trial included two different NMN dose groups [41]. The published studies covered the years between 2021 and 2025 and were performed in China (N = 2), Japan (N = 6), and the USA (N = 2). The included studies comprised a total of 349 participants, with 182 in the intervention groups and 167 in the placebo groups. All trials used a parallel and double-blind design. The specific NMN intervention protocol is detailed in Table S4. The daily dosage of NMN supplementation ranged from 250 mg to 1500 mg, and the intervention duration lasted from 4 to 12 weeks. Among the ten RCTs, six administered NMN in capsules, two used tablets, one used powder, and the remaining one did not report the form of oral NMN. The characteristics of the participants varied across the studies. Eight studies enrolled both females and males [26,33,34,35,36,38,40,41], while the remaining one study enrolled only females [37] or only males [39], respectively. One study was performed in individuals who were overweight or obese [38], another focused on patients with mild essential hypertension [26], while the remaining studies involved healthy populations with elevated blood pressure. According to the new categories for blood pressure, participants in four studies were classified as having stage 1 hypertension (SBP 130–139 mmHg or DBP 80–89 mmHg) [26,38,39,40], while those in the remaining six studies had elevated blood pressure (SBP 120–129 mmHg and DBP < 80 mmHg). Notably, no included trials enrolled participants with moderate to severe hypertension (SBP ≥ 140 mmHg or DBP ≥ 90 mmHg), indicating that the study population was predominantly composed of individuals with early-stage blood pressure elevation rather than established hypertension.

### 3.3. Risk of Bias Assessment

**Figure 2:**
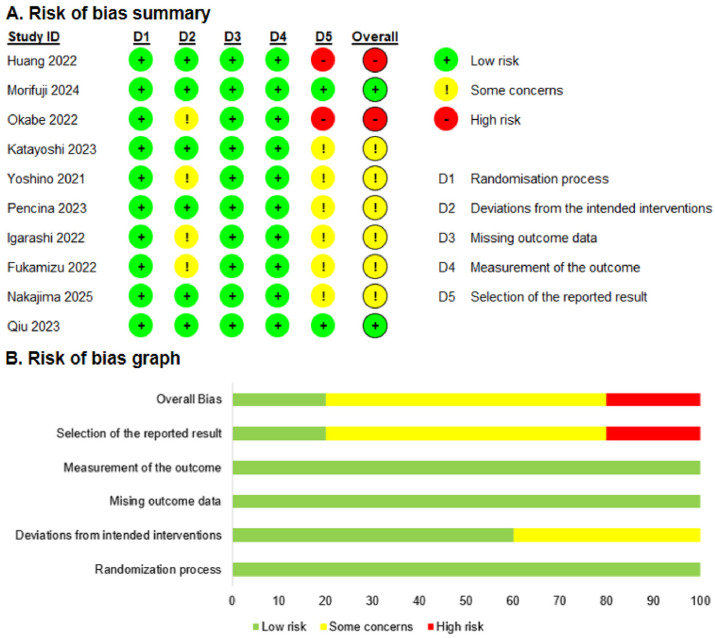
Risk of bias assessment of the included studies. (**A**). Risk of bias summary [26,33,34,35,36,37,38,39,40,41]. (**B**). Risk of bias graph.

The quality of all included RCTs was evaluated using the Cochrane RoB 2.0 tool (The Cochrane Collaboration, London, UK). As illustrated in Figure 2 and Table S5, two studies were classified as high quality [26,34], six studies raised some concerns [36,37,38,39,40,41], and the remaining two studies were assessed as poor quality [33,35]. The majority of the risk of bias was largely attributed to deviations from the intended interventions and the selection of the reported outcomes.

### 3.4. Effects of NMN Supplementation on Resting Blood Pressure

**Figure 3:**
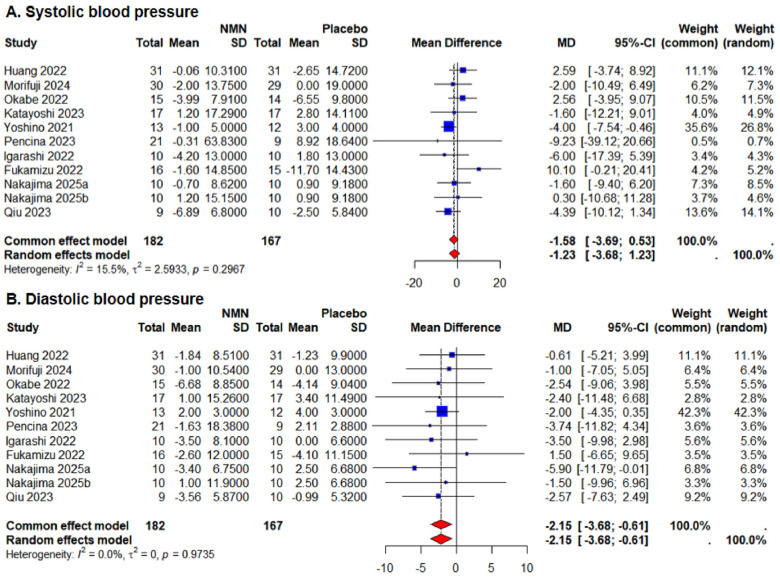
Meta-analysis of the effects of NMN supplementation on systolic blood pressure [26,33,34,35,36,37,38,39,40,41] (**A**) and diastolic blood pressure [26,33,34,35,36,37,38,39,40,41] (**B**). Nakajima 2025a [41]: the dosage of NMN was 750 mg per day; Nakajima 2025b [41]: the dosage of NMN was 1500 mg per day.

The comprehensive effects of oral NMN supplements on blood pressure are presented in Figure 3. When compared with the placebo, the common-effects model indicated that NMN supplementation was associated with a reduction of 2.15 mmHg in DBP (95% CI: −3.68 to −0.61), without observed heterogeneity (*I*^2^ = 0.0%). Although a slight reduction in SBP (WMD: −1.58 mmHg; 95% CI: −3.69 to 0.53; *I*^2^ = 15.5%) was observed after NMN supplementation, the common-effects model indicated that this decrease was not statistically significant.

As shown in Table 2, subgroup analyses revealed that significant reductions in DBP were not observed in the following subgroups: participants younger than 60 years old (WMD: −2.15 mmHg; 95% CI: −4.51 to 0.21), those with a BMI of 25 kg/m^2^ or greater (WMD: −1.84 mmHg; 95% CI: −3.87 to 0.19), interventions lasting less than 10 weeks (WMD: −2.23 mmHg; 95% CI: −4.73 to 0.28), daily NMN dosage exceeding 300 mg (WMD: −2.23 mmHg; 95% CI: −4.73 to 0.28), and baseline DBP of 80 mmHg or greater (WMD: −2.34 mmHg; 95% CI: −5.62 to 0.93). Notably, subgroup analyses suggested that NMN supplementation led to a statistically significant but exploratory reduction in SBP among participants aged 60 years and older (WMD: −3.94 mmHg; 95% CI: −7.06 to −0.82), as well as in studies conducted in non-Asian countries (WMD: −4.07 mmHg; 95% CI: −7.58 to −0.56).

### 3.5. Adverse Events and Safety

Six RCTs reported mild adverse events in both the NMN supplementation and the placebo groups [33,34,35,38,40,41]. The most frequently reported adverse events were minor gastrointestinal reactions, such as diarrhea and abdominal pain, all of which were resolved spontaneously and were not related to NMN supplementation. One study conducted by Okabe et al. reported additional side effects, including fever, joint pain, muscle pain, and fatigue; however, these events were not attributed to NMN supplementation [35]. The remaining four studies did not observe any adverse events in either group.

### 3.6. Sensitivity Analyses

Considering the differences observed in the subgroup analyses, sensitivity analyses were performed by sequentially removing each study to assess its impact on the overall effect size (Figure S1). The removal of any individual study did not significantly affect the overall effect size for either SBP or DBP following NMN supplementation, indicating the stability of the results. To further address the potential impact of methodological quality, a post hoc sensitivity analysis was conducted by excluding the two trials with a high risk of bias [33,35]. Using the same common-effects model (Figure S2), this exclusion resulted in enhanced and statistically significant reductions for both DBP (WMD: −2.32 mmHg; 95% CI: −4.00 to −0.65; *I*^2^ = 0.0%) and, notably, for SBP in the overall adult population (WMD: −2.73 mmHg; 95% CI: −5.11 to −0.35; *I*^2^ = 0.0%), in contrast to the non-significant original SBP result (WMD = −1.58 mmHg). The complete elimination of heterogeneity (*I*^2^ = 0.0%) in both analyses after excluding these studies suggests that study quality was the main source of the mild heterogeneity observed in the original SBP analysis.

### 3.7. Publication Bias

**Figure 4:**
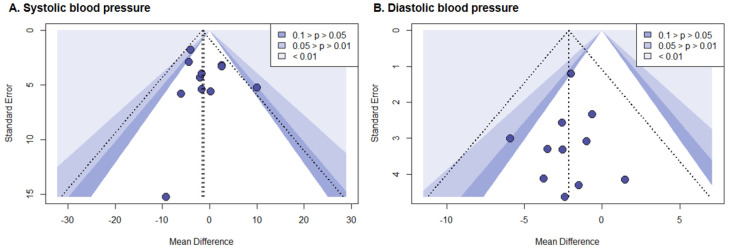
Publication bias assessment for systolic blood pressure (**A**) and diastolic blood pressure (**B**) using contour-enhanced funnel plots.

Begg’s rank correlation test and Egger’s linear regression analysis found no evidence of publication bias for SBP or DBP (all *p* > 0.05). As shown in Figure 4, the available data did not provide adequate evidence for the presence of asymmetry in the funnel plot, and no discernible outliers were observed for either SBP or DBP.

## 4. Discussion

The present meta-analysis reviewed ten RCTs with 11 intervention arms to comprehensively evaluate the effects of oral NMN supplements on resting blood pressure in adults with elevated blood pressure. The present findings suggested that NMN supplementation may be associated with a statistically significant but small reduction in resting DBP (*p* = 0.006). However, no significant effect on resting SBP (*p* > 0.05) was observed in the general adult population. Notably, subgroup analyses tentatively revealed a statistically significant decrease (*p* < 0.05) in resting SBP among participants aged 60 years and older, as well as in studies conducted in non-Asian countries.

To the best of our knowledge, this meta-analysis is the first to clarify whether resting blood pressure is improved by oral NMN supplements. Our systematic review adhered to standardized procedures, ensuring methodological rigor by following the PRISMA framework and using the Cochrane RoB 2.0 tool to assess the quality of the included RCTs. Although all studies employed a parallel and double-blind design, two trials were evaluated as having a high risk of bias due to selective reporting of results [33,35]. The study conducted by Huang et al. exhibited a relatively high risk of detection bias, primarily due to incomplete outcome reporting, as it presented only the significance levels of change values [33]. Similarly, the study conducted by Okabe et al. was also rated as poor quality because of selective reporting bias, specifically the omission of a longitudinal time-by-treatment interaction analysis [35]. Therefore, although these results provide preliminary suggestive evidence of an association, the presence of these specific reporting biases means that the pooled findings should be interpreted with considerable caution. The results of sensitivity analysis indicated that the removal of one of the two RCTs did not significantly affect the overall findings, suggesting that the effect size remained stable. Furthermore, a supplementary analysis of combined exclusion of these two high-risk bias studies revealed that their inclusion diluted the actual blood pressure-lowering effect of NMN. Exclusion of the high-risk studies resulted in a significant SBP reduction that was not evident in the primary analysis, a marginal improvement in the DBP effect size, and complete resolution of the mild heterogeneity previously observed in the SBP pooled estimate. This finding indicated that the selective reporting bias in the two studies [33,35] may have led to an underestimation of NMN’s efficacy on blood pressure. Therefore, further research should prioritize long-term, large-scale, and high-quality RCTs specifically designed to confirm NMN’s clinical utility.

The present study found a statistical reduction of 2.15 mmHg in resting DBP following NMN supplementation in adults with elevated blood pressure levels; however, no significant effects were observed in resting SBP. It is critical to note that reductions as minimal as 2 mmHg in resting DBP may be clinically meaningful in the context of population-level cardiovascular risk prevention [42], as they are associated with a 6% decrease in the risk of coronary heart disease and a 15% decrease in the risk of stroke. Compared to other non-pharmacological interventions, NMN supplementation exhibited a comparable and promising hypotensive effect in the management of early-stage blood pressure. Traditional aerobic exercise training resulted in a significant reduction in DBP, with a decrease of 2.53 mmHg following an exercise intervention lasting two weeks or longer [43]. Furthermore, the Dietary Approach to Stop Hypertension (DASH) diet—a dietary pattern rich in fruits, vegetables, whole grains, and low-fat dairy products—was found to produce a significant decrease in DBP of 2.60 mmHg after a duration of 2 to 24 weeks [44]. However, given that the included RCTs were smaller in scale, shorter in duration, and primarily focused on blood pressure as a secondary outcome, caution is warranted when interpreting the hypotensive effect of NMN. Subgroup analyses further revealed a more substantial reduction of 3.94 mmHg in resting SBP among participants aged 60 years and older with a baseline BP of 126.94/76.96 mmHg versus 125.04/77.33 mmHg in younger adults, as well as a reduction of 4.07 mmHg in studies conducted in non-Asian countries. The literature has demonstrated that the extent of blood pressure reduction is semilogarithmically correlated with the incidence of cardiovascular outcomes [45]. However, this epidemiological association must be interpreted cautiously in the context of the current study population. The participants were predominantly individuals with early-stage blood pressure elevation, and the BP reduction observed here may not translate to the same degree of individual-level clinical benefit as in patients with established hypertension. The modest effect size is largely constrained by the baseline blood pressure status, as the potential for blood pressure reduction is limited in individuals without severe hypertension. Its clinical significance of NMN supplementation at the individual level remains uncertain. Thus, the clinical relevance of this mild reduction should not be overstated and requires verification in long-term studies targeting diverse blood pressure subgroups.

Furthermore, according to the new categories for blood pressure [21], participants in ten included studies exhibited elevated blood pressure with four studies specifically focusing on individuals with stage 1 hypertension. Our results exhibited no heterogeneity and were unaffected by publication bias. Subgroup analyses indicated that NMN supplementation resulted in a moderate reduction of 3.94 mmHg in resting SBP among participants aged 60 years and older. These findings provide preliminary and suggestive evidence that NMN may serve as a possible avenue for blood pressure management in this population. Considering that only four RCTs were included, more RCTs are necessary to further explore the antihypertensive effects of NMN supplementation in individuals aged 60 years and older. Additionally, the present subgroup analyses revealed that NMN supplementation significantly reduced resting SBP in adults from non-Asia regions (mean baseline BP: 128.44/76.64 mmHg) and resting DBP in adults from Asia regions (mean baseline BP: 125.27/77.19 mmHg). These differences may be attributed to genetic background, varying dietary patterns and environmental factors [46]. This finding highlights a promising direction for future research integrating genetic analysis with epidemiological data to enable more targeted interventions. However, these results should be interpreted cautiously, as the observed effect in non-Asian populations was based on only two studies with limited statistical power, precluding definitive conclusions. These preliminary signals are hypothesis-generating, underscoring the need for large-scale RCTs specifically designed to validate geographic and ethnic differences.

The biological plausibility for these effects is rooted in NMN’s role as a precursor to NAD^+^ [47], which is produced via the salvage pathway in NAD^+^ biosynthesis [47,48]. Both animal and human studies have demonstrated that increased NAD^+^ levels can enhance endothelial-mediated vasorelaxation and mitigate arterial stiffness [15,49,50,51]. Consistent with this, animal studies have shown that NMN supplementation can mitigate NAD^+^ exhaustion, alleviate endothelial dysfunction and large elastic artery stiffening by activating SIRT1 [15,26]. These findings from animal models provide a compelling mechanistic rationale for the potential blood pressure-lowering effects of NMN, particularly in the context of vascular aging, which aligns with our subgroup observation of greater SBP reduction in older adults. However, it is important to emphasize that direct human evidence for these mechanisms remains limited. While a small number of human studies have reported that NAD^+^ precursors can improve vascular function, few have directly investigated whether NMN lowers blood pressure through the specific pathways identified in animal models. Therefore, while the preclinical evidence provides strong biological plausibility, the pathways through which NMN may exert its effects in humans remain largely hypothetical and require confirmation in future mechanistic trials.

Nonetheless, the optimal and maximum safe dosage of NMN for long-term administration has not been fully established, as the required clinical and toxicological studies remain incomplete [10]. In the ten included RCTs, the dosage of oral NMN supplements varied from 250 to 1500 mg per day. Most studies reported only mild, transient gastrointestinal side effects that did not present a serious risk to the health of the participants. Furthermore, these events were not associated with NMN supplements and resolved spontaneously. Additionally, the study conducted by Okabe et al. identified adverse reactions, such as fever, joint pain, muscle pain, or fatigue, that may have been linked to COVID-19 vaccination rather than the NMN intervention itself [35]. Given the growing interest in NMN as a supplement for health, addressing a long-term safe dosage should be a primary focus for future research.

The present meta-analysis has several limitations. First, the methodological quality of the included trials was suboptimal, as two RCTs were classified as high risk of bias due to the selective reporting of results [33,35]. Therefore, the findings from this meta-analysis should be interpreted with caution. Second, despite the low heterogeneity in the present meta-analysis, the limited number of included trials and their small sample sizes with varying individuals restricted the strength and generalizability of the findings, as well as made it difficult to conduct sex-stratified analysis. It is important to investigate whether sex differences exist in the effects of NMN supplementation on blood pressure in the future research. Worth highlighting is that the subgroup analyses for adults aged 60 years and older and non-Asian populations are notably underpowered, with only 4 intervention arms and 2 studies included. This insufficient statistical power suggests that the significant SBP reductions observed in these groups may be chance occurrences and thus lack robustness. Third, all included studies were conducted exclusively in North America and Asia, resulting in a notable lack of geographical diversity. This limits the generalizability of the findings to global populations with distinct ethnic backgrounds, dietary patterns, and genetic characteristics. Furthermore, dietary habits were not systematically controlled or reported across the included studies, and intrinsic NMN intake from natural food sources was not documented. Fourth, most studies within the included RCTs considered changes in blood pressure as secondary or exploratory outcomes. This may lead to insufficient statistical power for detecting small but potentially clinically meaningful changes in blood pressure, potentially resulting in either underestimation or overestimation of the effects of NMN intervention on blood pressure. Lastly, it is important to note that the included trials primarily enrolled individuals with elevated blood pressure or stage 1 hypertension. Consequently, the present evidence may be more applicable to assessing the potential of NMN in early-stage blood pressure management. Whether NMN has protective effects on patients with hypertension is worth discussing. To address these limitations, large-scale clinical trials with high-quality randomized designs are essential. Such trials should be performed across various geographical areas and include diverse targeted populations with longer intervention durations to accurately determine the effects of NMN supplementation on blood pressure management across different sexes.

## 5. Conclusions

In conclusion, this meta-analysis provides preliminary and suggestive evidence that NMN supplementation may exert a modest effect on lowering DBP and may serve as a viable avenue for blood pressure management in adults with elevated blood pressure, particularly for SBP among individuals aged 60 and older. However, this small reduction has not been definitively shown to have meaningful clinical physiological significance and therefore warrants future investigation. Additionally, due to the limited number of included RCTs and the fact that blood pressure was often a secondary outcome in the included studies, these findings should be interpreted with caution. Notably, as the majority of participants had elevated blood pressure without a formal clinical diagnosis of hypertension, the efficacy of NMN supplementation in individuals with clinically diagnosed hypertension remains unknown, and for the general healthy population, more appropriate and evidence-based targeted tools for blood pressure prevention are currently available. Furthermore, given the existence of numerous well-established and evidence-based other non-pharmaceutical interventions [21,43,44] for elevated blood pressure, additional research is required to explore not merely whether NMN supplementation produces effects, but how its value compares to these established approaches. Therefore, further research should prioritize long-term, large-scale, and high-quality RCTs specifically designed to confirm its clinical utility and evaluate the optimal dosage and long-term safety of NMN for blood pressure management across diverse populations, including individuals with elevated blood pressure and those with established hypertension.

## Figures and Tables

**Table 1:** Main characteristic of the included trials and participants in this meta-analysis.

Author (Year, Country)	Study Design	Study Population	Sample Size *	Male (%)	Age * (years)	BMI * (kg/m^2^)	Duration	Intervention	Baseline BP * (mmHg)	Results
Huang [33] (2022, China)	RA, P, DB	Healthy subjects with elevated blood pressure	31/31	45.2	47.8 ± 6.6 vs. 47.2 ± 6.6	25.3 ± 2.3 vs. 24.7 ± 2.4	60 days	NMN (300 mg/d capsule) vs. placebo	124.26 ± 14.73/78.65 ± 8.76 vs. 128.90 ± 15.84/79.06 ± 7.46	SBP ↔; DBP ↔
Morifuji [34] (2024, Japan)	RA, P, DB	Healthy subjects with elevated blood pressure	30/29	60.0	69.0 ± 3.0 vs. 69.0 ± 3.0	22.4 ± 2.6 vs. 22.6 ± 3.6	12 weeks	NMN (250 mg/d capsule) vs. placebo	124.00 ± 12.00/75.00 ± 10.00 vs. 124.00 ± 19.00/77.00 ± 13.00	SBP ↔; DBP ↔
Okabe [35] (2022, Japan)	RA, P, DB	Healthy subjects with elevated blood pressure	15/14	26.7	42.9 ± 12.0 vs. 43.9 ± 9.9	21.3 ± 2.5 vs. 21.1 ± 2.1	12 weeks	NMN (250 mg/d tablet) vs. placebo	121.10 ± 6.20/74.30 ± 8.90 vs. 123.30 ± 10.70/74.50 ± 7.50	SBP ↔; DBP ↔
Katayoshi [36] (2023, Japan)	RA, P, DB	Healthy subjects with elevated blood pressure	17/17	38.9	48.1 ± 5.4 vs. 47.9 ± 5.5	21.7 ± 4.2 vs. 21.5 ± 2.2	12 weeks	NMN (250 mg/d capsule) vs. placebo	118.00 ± 16.20/69.80 ± 15.00 vs. 124.30 ± 15.00/76.00 ± 12.40	SBP ↔; DBP ↔
Yoshino [37] (2021, USA)	RA, P, DB	Postmenopausal women with elevated blood pressure	13/12	0	62.0 ± 4.0 vs. 61.0 ± 5.0	33.7 ± 1.4 vs. 33.4 ± 1.0	10 weeks	NMN (250 mg/d capsule) vs. placebo	126.00 ± 5.00/73.00 ± 3.00 vs. 128.00 ± 4.00/73.00 ± 3.00	SBP ↔; DBP ↔
Pencina [38] (2023, USA)	RA, P, DB	Overweight or obese adults with stage 1 hypertension	21/9	53.3	60.9 ± 8.9 vs. 64.3 ± 7.6	29.1 ± 3.6 vs. 29.5 ± 3.8	4 weeks	NMN (1000 mg/d tablet) vs. placebo	128.00 ± 14.60/80.00 ± 8.91 vs. 133.60 ± 14.90/78.90 ± 6.86	SBP ↔; DBP ↓
Igarashi [39] (2022, Japan)	RA, P, DB	Healthy subjects with stage 1 hypertension	10/10	100	71.1 ± 3.9 vs. 71.8 ± 6.1	23.7 ± 1.3 vs. 24.8 ± 1.2	12 weeks	NMN (250 mg/d) vs. placebo	135.90 ± 18.90/85.20 ± 13.60 vs. 127.00 ± 18.20/81.20 ± 10.90	SBP ↔; DBP ↔
Fukamizu [40] (2022, Japan)	RA, P, DB	Healthy subjects with stage 1 hypertension	16/15	45.2	35.1 ± 7.0 vs. 35.7 ± 7.2	22.9 ± 2.7 vs. 22.1 ± 3.3	4 weeks	NMN (1250 mg/d powder) vs. placebo	127.20 ± 14.90/80.40 ± 11.90 vs. 128.80 ± 14.90/80.80 ± 11.10	SBP ↔; DBP ↔
Nakajima [41] (2025, Japan)	RA, P, DB	Healthy subjects with elevated blood pressure	10/10	45.0	46.9 ± 7.7 vs. 49.7 ± 7.8	22.4 ± 2.7 vs. 22.8 ± 2.2	4 weeks	NMN (750 mg/d capsule) vs. placebo	120.40 ± 9.70/75.60 ± 6.80 vs. 121.10 ± 8.70/73.40 ± 7.30	SBP ↔; DBP ↔
	RA, P, DB	Healthy subjects with elevated blood pressure	10/10	45.0	48.9 ± 8.8 vs. 49.7 ± 7.8	22.7 ± 4.2 vs. 22.8 ± 2.2	4 weeks	NMN (1500 mg/d capsule) vs. placebo	117.00 ± 15.30/72.30 ± 12.50 vs. 121.10 ± 8.70/73.40 ± 7.30	SBP ↔; DBP ↔
Qiu [26] (2023, China)	RA, P, DB	Mild essential hypertensive patients	9/10	47.4	46.0 ± 12.9 vs. 46.7 ± 11.2	22.9 ± 3.4 vs. 22.9 ± 1.9	6 weeks	NMN (800 mg/d capsule) vs. placebo	136.22 ± 7.61/82.89 ± 6.57 vs. 136.40 ± 5.78/82.90 ± 5.51	SBP ↓; DBP ↔

Note: * Intervention group vs. placebo group. “↓” refers to a decrease; “↔” refers to no change. RA, randomized; P, parallel; DB, double blind; BMI, body mass index; BP, blood pressure.

**Table 2:** Subgroup analysis of NMN supplementation on blood pressure according to trials and participant characteristics.

Group	N	Net Change (95% CI)	*I*^2^ (%)	*p*-Heterogeneity
Systolic blood pressure				
Total	11	−1.58 (−3.69, 0.53)	15.5	0.297
Age				
<60 years	7	0.40 (−2.47, 3.26)	18.5	0.289
≥60 years	4	−3.94 (−7.06, −0.82)	0	0.930
Baseline BMI				
<25 kg/m^2^	8	−0.76 (−3.66, 2.14)	10.3	0.350
≥25 kg/m^2^	3	−2.50 (−5.57, 0.57)	40.7	0.185
Location				
Asia	9	−0.18 (−2.82, 2.46)	8.0	0.369
Non-Asia	2	−4.07 (−7.58, −0.56)	0	0.734
Duration				
<10 weeks	6	−0.09 (−3.41, 3.23)	29.7	0.212
≥10 weeks	5	−2.59 (−5.33, 0.14)	0	0.492
NMN dose				
<300 mg/d	5	−2.59 (−5.33, 0.14)	0	0.492
≥300 mg/d	6	−0.09 (−3.41, 3.23)	29.7	0.212
Baseline SBP				
<130 mmHg	8	−0.89 (−3.21, 1.43)	28.1	0.204
≥130 mmHg	3	−4.84 (−9.89, 0.20)	0	0.929
Diastolic blood pressure				
Total	11	−2.15 (−3.68, −0.61)	0	0.974
Age				
<60 years	7	−2.15 (−4.51, 0.21)	0	0.830
≥60 years	4	−2.14 (−4.15, −0.13)	0	0.926
Baseline BMI				
<25 kg/m^2^	8	−2.55(−4.88, −0.22)	0	0.920
≥25 kg/m^2^	3	−1.84(−3.87, 0.19)	0	0.777
Location				
Asia	9	−2.15 (−4.23, −0.07)	0	0.926
Non-Asia	2	−2.14 (−4.40, 0.12)	0	0.685
Duration				
<10 weeks	6	−2.23 (−4.73, 0.28)	0	0.707
≥10 weeks	5	−2.10 (−4.03, −0.16)	0	0.987
NMN dose				
<300 mg/d	5	−2.10 (−4.03, −0.16)	0	0.987
≥300 mg/d	6	−2.23 (−4.73, 0.28)	0	0.707
Baseline DBP				
<80 mmHg	7	−2.09 (−3.82, −0.36)	0	0.902
≥80 mmHg	4	−2.34 (−5.62, 0.93)	0	0.777

## Data Availability

The original contributions presented in this study are included in the article/Supplementary Material. Further inquiries can be directed to the corresponding authors.

## Supplementary Materials

The following supporting information can be downloaded at https://www.mdpi.com/article/10.3390/nu18060890/s1: Table S1: Preferred Reporting Items for Systematic Reviews and Meta-Analyses (PRISMA); Table S2: Search terms employed for screening in the literature search; Table S3: PICOS criteria for inclusion and exclusion of studies; Table S4: Characteristics of the NMN intervention mode in included trials; Table S5: Risk of bias assessment of included studies; Table S6: Baseline blood pressure characteristics of included studies across subgroups; Figure S1: Sensitivity analysis of systolic blood pressure (A) and diastolic blood pressure (B); Figure S2: Forest plot of the effect of NMN supplementation on systolic blood pressure (A) and diastolic blood pressure (B) after excluding studies with high risk of bias.

## Abbreviations

The following abbreviations are used in this manuscript:

BMI	Body mass index
BP	Blood pressure
CI	Confidence Interval
DBP	Diastolic blood pressure
PICOS	Population, Intervention, Comparison, Outcome, Study
PRISMA	Preferred Reporting Items for Systematic Reviews and Meta-Analyses
PROSPERO	International Prospective Register of Systematic Reviews
RCTs	Randomized controlled trial
SBP	Systolic blood pressure
SD	Standard deviation
WMD	Weighted mean difference
