# Supplementary material for: Effects of Nicotinamide Mononucleotide Supplementation on Blood Pressure: A Systematic Review and Meta-Analysis of Randomized Controlled Trials
Source: Nutrients. 2026 Mar 11;18(6):890. doi: 10.3390/nu18060890 (PMC13028934; doi:10.3390/nu18060890)

Supplementary Materials

Table S1 Preferred Reporting Items for Systematic Reviews and Meta-Analyses (PRISMA).

Table S2 Search terms employed for screening in the literature search.

Table S3 PICOS criteria for inclusion and exclusion of studies.

Table S4 Characteristics of the NMN intervention mode in included trials.

Table S5 Risk of bias assessment of included studies.

Table S6 Baseline blood pressure characteristics of included studies across subgroups.

Figure S1 Sensitivity analysis of systolic blood pressure (A) and diastolic blood pressure (B). Nakajima 2025a: NMN intake 750 mg/d; Nakajima 2025b: NMN intake 1500 mg/d.

Figure S2 Forest plot of the effect of NMN supplementation on systolic blood pressure (A) and diastolic blood pressure (B) after excluding studies with high risk of bias. Nakajima 2025a: NMN intake 750 mg/d; Nakajima 2025b: NMN intake 1500 mg/d.

Table S1. Preferred Reporting Items for Systematic Reviews and Meta-Analyses (PRISMA).

Section and Topic	Item #	Checklist item	Location where item is reported
TITLE			
Title	1	Identify the report as a systematic review.	Title page
ABSTRACT			
Abstract	2	See the PRISMA 2020 for Abstracts checklist.	Abstract
INTRODUCTION			
Rationale	3	Describe the rationale for the review in the context of existing knowledge.	Introduction: paragraph 1-3
Objectives	4	Provide an explicit statement of the objective(s) or question(s) the review addresses.	Introduction: paragraph 4
METHODS			
Eligibility criteria	5	Specify the inclusion and exclusion criteria for the review and how studies were grouped for the syntheses.	Material and methods: study selection
Information sources	6	Specify all databases, registers, websites, organisations, reference lists and other sources searched or consulted to identify studies. Specify the date when each source was last searched or consulted.	Material and methods: data sources and search strategy
Search strategy	7	Present the full search strategies for all databases, registers and websites, including any filters and limits used.	Material and methods: data sources and search strategy<br>Table S2
Selection process	8	Specify the methods used to decide whether a study met the inclusion criteria of the review, including how many reviewers screened each record and each report retrieved, whether they worked independently, and if applicable, details of automation tools used in the process.	Material and methods: study selection;<br>Material and methods: data extraction;<br>Figure 1<br>Table S3
Data collection process	9	Specify the methods used to collect data from reports, including how many reviewers collected data from each report, whether they worked independently, any processes for obtaining or confirming data from study investigators, and if applicable, details of automation tools used in the process.	Material and methods: data extraction
Data items	10a	List and define all outcomes for which data were sought. Specify whether all results that were compatible with each outcome domain in each study were sought (e.g. for all measures, time points, analyses), and if not, the methods used to decide which results to collect.	Material and methods: data extraction
	10b	List and define all other variables for which data were sought (e.g. participant and intervention characteristics, funding sources). Describe any assumptions made about any missing or unclear information.	Material and methods: data extraction
Study risk of bias assessment	11	Specify the methods used to assess risk of bias in the included studies, including details of the tool(s) used, how many reviewers assessed each study and whether they worked independently, and if applicable, details of automation tools used in the process.	Materials and methods: quality assessment
Effect measures	12	Specify for each outcome the effect measure(s) (e.g. risk ratio, mean difference) used in the synthesis or presentation of results.	Materials and methods: data syntheses and statistical analyses

Section and Topic	Item #	Checklist item	Location where item is reported
Synthesis methods	13a	Describe the processes used to decide which studies were eligible for each synthesis (e.g. tabulating the study intervention characteristics and comparing against the planned groups for each synthesis (item #5)).	Material and methods: study selection; Table 1
	13b	Describe any methods required to prepare the data for presentation or synthesis, such as handling of missing summary statistics, or data conversions.	Materials and methods: data extraction; Materials and methods: data syntheses and statistical analyses
	13c	Describe any methods used to tabulate or visually display results of individual studies and syntheses.	Materials and methods: data syntheses and statistical analyses
	13d	Describe any methods used to synthesize results and provide a rationale for the choice(s). If meta-analysis was performed, describe the model(s), method(s) to identify the presence and extent of statistical heterogeneity, and software package(s) used.	Materials and methods: data syntheses and statistical analyses
	13e	Describe any methods used to explore possible causes of heterogeneity among study results (e.g. subgroup analysis, meta-regression).	Materials and methods: data syntheses and statistical analyses
	13f	Describe any sensitivity analyses conducted to assess robustness of the synthesized results.	Materials and methods: data syntheses and statistical analyses
Reporting bias assessment	14	Describe any methods used to assess risk of bias due to missing results in a synthesis (arising from reporting biases).	Materials and methods: data syntheses and statistical analyses
Certainty assessment	15	Describe any methods used to assess certainty (or confidence) in the body of evidence for an outcome.	Materials and methods: data syntheses and statistical analyses
RESULTS			
Study selection	16a	Describe the results of the search and selection process, from the number of records identified in the search to the number of studies included in the review, ideally using a flow diagram.	Results: literature search; Figure 1
	16b	Cite studies that might appear to meet the inclusion criteria, but which were excluded, and explain why they were excluded.	Figure 1
Study characteristics	17	Cite each included study and present its characteristics.	Results: study characteristics Table 1 Table S4
Risk of bias in studies	18	Present assessments of risk of bias for each included study.	Results: risk of bias assessment; Figure 2
Results of individual studies	19	For all outcomes, present, for each study: (a) summary statistics for each group (where appropriate) and (b) an effect to estimate and its precision (e.g. confidence/credible interval), ideally using structured tables or plots.	Figure 3 Table 2
Results of syntheses	20a	For each synthesis, briefly summarise the characteristics and risk of bias among contributing studies.	Results: study characteristics; Results: risk of bias assessment;

Section and Topic	Item #	Checklist item	Location where item is reported
			Results: adverse events and safety
	20b	Present results of all statistical syntheses conducted. If meta-analysis was done, present for each the summary estimate and its precision (e.g. confidence/credible interval) and measures of statistical heterogeneity. If comparing groups, describe the direction of the effect.	Results: effects of NMN supplementation on resting blood pressure;<br>Figure 3<br>Table 2
	20c	Present results of all investigations of possible causes of heterogeneity among study results.	Results: effects of NMN supplementation on resting blood pressure
	20d	Present results of all sensitivity analyses conducted to assess the robustness of the synthesized results.	Results: sensitivity analyses
Reporting biases	21	Present assessments of risk of bias due to missing results (arising from reporting biases) for each synthesis assessed.	Results: risk of bias assessment;<br>Figure S2<br>Table S5
Certainty of evidence	22	Present assessments of certainty (or confidence) in the body of evidence for each outcome assessed.	Results: risk of bias assessment;<br>Results: Sensitivity analyses<br>Results: Publication bias
DISCUSSION			
Discussion	23a	Provide a general interpretation of the results in the context of other evidence.	Discussion: paragraph 1
	23b	Discuss any limitations of the evidence included in the review.	Discussion: paragraph 2, 6
	23c	Discuss any limitations of the review processes used.	Discussion: paragraph 7
	23d	Discuss implications of the results for practice, policy, and future research.	Discussion: paragraph 7
OTHER INFORMATION			
Registration and protocol	24a	Provide registration information for the review, including register name and registration number, or state that the review was not registered.	CRD42025635763
	24b	Indicate where the review protocol can be accessed, or state that a protocol was not prepared.	https://www.crd.york.ac.uk/PROSPERO/
	24c	Describe and explain any amendments to information provided at registration or in the protocol.	N/A
Support	25	Describe sources of financial or non-financial support for the review, and the role of the funders or sponsors in the review.	Funding Statement
Competing interests	26	Declare any competing interests of review authors.	Conflicts of Interest
Availability of data, code and other materials	27	Report which of the following are publicly available and where they can be found: template data collection forms; data extracted from included studies; data used for all analyses; analytic code; any other materials used in the review.	Data Availability Statement

Table S2 Search terms employed for screening in the literature search.

Database	Search terms
PubMed/MEDLINE	("Nicotinamide mononucleotide" OR "Mononucleotide nicotinamide" OR "NMN" OR "NAD") AND ("blood pressure" OR "BP" OR "hypertension") AND ("clinical trials" OR "RCT" OR "intervention" OR "trial" OR "randomized" OR "crossover" OR "placebo")
Scopus	ALL (nicotinamide mononucleotide OR nmn OR mononucleotide nicotinamide OR NAD) AND ALL (clinical trials OR rct OR intervention OR trial OR randomized OR crossover OR placebo) AND ALL (blood pressure OR BP OR hypertension)
Web of Science	(ALL = (nicotinamide mononucleotide) OR ALL = (mononucleotide nicotinamide) OR ALL=(NMN) OR ALL=(NAD)) AND (ALL = (blood pressure) OR ALL=(BP) OR ALL=(hypertension)) AND (ALL = (clinical trials) OR ALL=(RCT) OR ALL=(intervention) OR ALL=(trial) OR ALL=(randomized) OR ALL=(crossover) OR ALL=(placebo))
EBSCO	(nicotinamide mononucleotide OR mononucleotide nicotinamide OR NMN OR NAD) AND (blood pressure OR BP OR hypertension) AND (clinical trials OR RCT OR intervention OR trial OR randomized OR crossover OR placebo)

Table S3 PICOS criteria for inclusion and exclusion of studies.

Parameter	Inclusion criteria	Exclusion criteria
Participants	All sexes, races and ethnicities; adults aged 18 years and older	Animal studies and studies among children
Interventions	Studies with oral NMN supplements, intervention duration ≥ 4 weeks	Studies without oral NMN supplements, or involving NMN supplements in combination with other dietary supplements; intervention duration < 4 weeks
Comparisons	Placebo or without oral NMN supplements	Lack of comparison group or placebo-controlled group
Outcomes	Studies providing sufficient data to calculate the net changes in blood pressure along with their corresponding standard deviation both pre- and post-intervention	Not calculating the net changes in blood pressure along with their corresponding standard deviation both pre- and post-intervention
Study design	Randomized controlled trials with parallel or crossover design	Non-randomized studies or non-primary studies, including reviews, letters, conference abstracts, case reports, and observational studies (cross-sectional studies, cohort studies, and case-control studies)

Table S4 Characteristics of the NMN intervention mode in included trials.

Author (Year, country)	NMN form	NMN dosage	Dosage frequency	Duration of intervention
Huang (2022, China)	capsule	300 mg/d	Once daily	60 days
Morifuji (2024, Japan)	capsule	250 mg/d	Twice daily	12 weeks
Okabe (2022, Japan)	tablet	250 mg/d	Twice daily	12 weeks
Katayoshi (2023, Japan)	capsule	250 mg/d	Twice daily	12 weeks
Yoshino (2021, USA)	capsule	250 mg/d	Once daily	10 weeks
Pencina (2023, USA)	tablet	1000 mg/d	Twice daily	4 weeks
Igarashi (2022, Japan)	/	250 mg/d	Once daily	12 weeks
Fukamizu (2022, Japan)	powder	1250 mg/d	Once daily	4 weeks
Nakajima	capsule	750 mg/d	/	4 weeks
(2025, Japan)	capsule	1500 mg/d	/	4 weeks
Qiu (2023, China)	capsule	800 mg/d	Once daily	6 weeks

Table S5 Risk of bias assessment of included studies

Bias	Author's judgement	Support for judgement
**Huang 2022**		
Randomization process	Low	"These 66 subjects were randomized to either the NMN arm or the placebo arm according to the randomization scheme in a 1:1 ratio."
Deviations from intended interventions	Low	"None of the subjects were excluded from the efficacy analysis because of any adverse event or any violation."
Missing outcome data	Low	Outcome data were available for nearly all randomized participants.
Measurement of the outcome	Low	The method of outcome measurement was unlikely to introduce bias between groups.
Selection of the reported result	High	"Due to random nature of treatment allocation, after unblinding 31 subjects were found to be on active arm and 35 to be on the placebo arm. Hence 31 subjects from each group were selected for analysis and analysis was performed on total 62 subjects."
Overall bias	High	
**Morifuji 2024**		
Randomization process	Low	"60 participants were randomly allocated into two groups (NMN group and placebo group). The number of steps in the stepping test was used as an allocation factor."
Deviations from intended interventions	Low	Participants were asked to keep a diary during the study.<br>The study was double-blind with indistinguishable capsules.<br>Participants were restricted from starting new supplements during the study.
Missing outcome data	Low	A few participants were absent at follow-up due to illness or injury. All randomized subjects were included in safety analysis, and the missing data were unlikely to bias the results.
Measurement of the outcome	Low	The method of outcome measurement was unlikely to introduce bias between groups.
Selection of the reported result	Low	Primary and secondary outcomes were pre-specified and reported with precise P-values and effect sizes. No evidence of selective reporting.
Overall bias	Low	
**Okabe 2022**		
Randomization process	Low	"Participants were randomly allocated to the NMN or the placebo group in a one-to-one ratio by C&C Qualitative Research Institute Inc. (Tokyo, Japan) based on the principal that the background and screening test results of participants were not biased between groups. The information of allocation was kept by C&C Qualitative Research Institute Inc., and the key open was done after all results were fixed."
Deviations from	Some	The analytical method used to estimate the effect of being assigned

intended interventions	concerns	to the intervention was not described; however, even without analyzing participants according to their randomized groups, it likely would not have substantially impacted the results.
Missing outcome data	Low	Outcome data were available for nearly all randomized participants.
Measurement of the outcome	Low	The method of outcome measurement was unlikely to introduce bias between groups.
Selection of the reported result	High	The time × treatment interaction effect was not examined by any statistical analyses.
Overall bias	High	
**Katayoshi 2023**		
Randomization process	Low	"An allocation controller at Orthomedico Inc. (Tokyo, Japan) randomly assigned subjects to two supplementation groups (NMN or placebo) by block random allocation. Information on allocation was not opened until the subjects for analysis were determined at a clinical meeting after test completion."
Deviations from intended interventions	Low	"Statistical analyses of the baseline characteristics of subjects were performed in an intention-to-treat population."<br>"The primary efficacy variables were statistically analyzed using a full-analysis-set population."
Missing outcome data	Low	"Following the 12-week intervention, one participant in each group was untraceable and was considered a dropout."<br>The reasons for the missing data were unrelated to the intervention, and the impact on the results was likely to be small.
Measurement of the outcome	Low	The method of outcome measurement was unlikely to introduce bias between groups.
Selection of the reported result	Some concerns	The pre-specified analysis plan was not reported.
Overall bias	Some concerns	
**Yoshino 2021**		
Randomization process	Low	"After all baseline studies were completed, participants were randomly assigned to 10 weeks of treatment with placebo or NMN by using a computer-generated randomization scheme stratified by BMI."<br>"The study was conducted in a double-blind fashion and both the participants and members of the research team directly involved with study participants, dispensing study supplements, and assessing study outcomes were blinded to group assignment until completion of the study."
Deviations from intended interventions	Some concerns	"Therefore, the data from 12 participants in the placebo group and 13 in the NMN group completed the study and are reported here."<br>A total of 33 participants were randomized after completing baseline testing.

Missing outcome data	Low	The restriction to completer analysis is unlikely to have introduced bias.
Measurement of the outcome	Low	The method of outcome measurement was unlikely to introduce bias between groups.
Selection of the reported result	Some concerns	The pre-specified analysis plan was not reported.
Overall bias	Some concerns	
**Pencina 2023**		
Randomization process	Low	"Eligible participants were randomized in a 2:1 ratio, stratified by sex, using concealed block randomization. The participants and study staff were masked; only the unblinded study biostatistician, the research pharmacist, and the Data and Safety Review Board had access to intervention assignment."
Deviations from intended interventions	Low	"All analyses were performed using intent-to-treat principle."
Missing outcome data	Low	Two randomized participants did not complete the study. Missing data were unreported to be related to the intervention, and core outcome data showed minimal loss.
Measurement of the outcome	Low	The method of outcome measurement was unlikely to introduce bias between groups.
Selection of the reported result	Some concerns	"NMN, NAD, and NAD metabolome, and safety endpoints were analyzed as absolute values as well as changes from baseline." However, other outcomes were only reported and analyzed as changes from baseline.
Overall bias	Some concerns	
**Igarashi 2022**		
Randomization process	Low	"After completion of the baseline investigations, participants were randomized to a 12-week supplementation of NMN or a placebo, with daily administration by a third party, C&C QUALITATIVE RESEARCH INSTITUTE INC (Tokyo, Japan); there were no significant differences in age, BMI, or SMI between the two groups. The allocation to the NMN or placebo group was also managed by C&C QUALITATIVE RESEARCH INSTITUTE INC until the end of the study. The participants and data collectors were blinded to the treatment. Once all participants completed the study, the randomization code was released."
Deviations from intended interventions	Some concerns	"at the 6-week visit, 11 participants each in the NMN and placebo groups received the other supplement owing to an error made by the supplier. According to the decision of the Ethics Committee of the University of Tokyo Hospital, we decided to exclude the data acquired from the 22 participants during the 12-week visit."

Missing outcome data	Low	The risk of bias due to missing outcome data was judged to be low, although data were obtained from only 50% of participants.
Measurement of the outcome	Low	The method of outcome measurement was unlikely to introduce bias between groups.
Selection of the reported result	Some concerns	Because of the errors in NMN or placebo allocation, pre-specified analysis plan was subject to changes after unblinding. However, the numerical results being assessed were probably not likely to have been selected.
Overall bias	Some concerns	
**Fukamizu 2022**		
Randomization process	Low	"Subjects were randomly assigned to two groups, placebo and NMN, using a random number table by the controller of Pharma Foods International Co., Ltd., who was not involved in the study. The controller sealed the allocation sheets and kept them in a sealed envelope until the data analysis was completed."<br>"In this study, there was no heterogeneity in the physical data of the subjects in either group."<br>"The selected subjects were randomly assigned to the placebo group or the NMN group by a staff member who was not involved in the study, using a random number table."
Deviations from intended interventions	Some concerns	"Subjects who consumed less than 80% of the test food and did not comply with the study instructions were excluded from the statistical analysis."
Missing outcome data	Low	"Since one subject withdrew for reasons unrelated to the study before the start of the study, the final number of subjects was 31." Outcome data were available for nearly all randomized participants.
Measurement of the outcome	Low	The method of outcome measurement was unlikely to introduce bias between groups.
Selection of the reported result	Some concerns	The pre-specified analysis plan was not reported.
Overall bias	Some concerns	
**Nakajima 2025**		
Randomization process	Low	"The participants were randomly allocated with stratified randomization to three groups (high-dose, low-dose and placebo) to ensure that there was no bias in age, sex or body mass index at screening by statistical analysis manager."
Deviations from intended interventions	Low	The test food intake rate for all participants was 100% and all participants ingested the test food as prescribed.
Missing outcome data	Low	Outcome data were available for nearly all randomized participants.

Measurement of the outcome	Low	The method of outcome measurement was unlikely to introduce bias between groups.
Selection of the reported result	Some concerns	The pre-specified analysis plan was not reported.
Overall bias	Some concerns	
**Qiu 2023**		
Randomization process	Low	“In total, 21 patients were enrolled and randomly assigned to the NMN (NMN10000 WRIGHT LIFE®) or lifestyle modification group for 30-day treatment using a computer-generated random list before the initiation of the trial.”
Deviations from intended interventions	Low	“Patients in NMN group received 800 mg NMN once daily and instructions about lifestyle modification; patients in the lifestyle modification group only improved their life styles. All the enrolled patients were confirmed whether they have followed the lifestyle instruction when finishing the trial.”<br>Adherence to the intervention was monitored throughout the study, with no major protocol deviations.
Missing outcome data	Low	Two participants withdrew prematurely from the trial, which resulted in 19 participants finishing the study.<br>Outcome data were available for nearly all randomized participants.
Measurement of the outcome	Low	The method of outcome measurement was unlikely to introduce bias between groups.
Selection of the reported result	Low	All prespecified outcomes were fully reported in the manuscript. There was no evidence of selective outcome reporting, such as omission, substitution, or selective reporting of only statistically significant results.
Overall bias	Low	

Table S6 Baseline blood pressure characteristics of included studies across subgroups.

Group	N of studies	Total	NMN intervention group	placebo group
Baseline SBP (mmHg)				
Overall	11	125.77 ± 14.16	124.81 ± 13.81	126.81 ± 14.51
Age				
< 60 years	7	125.04 ± 13.80	123.24 ± 13.94	126.85 ± 13.47
≥ 60 years	4	126.94 ± 14.70	127.09 ± 13.37	126.74 ± 16.31
Baseline BMI				
< 25 kg/m^2	8	124.91 ± 14.41	124.25 ± 14.13	125.59 ± 14.72
≥ 25 kg/m^2	3	127.46 ± 13.55	125.82 ± 13.26	129.51 ± 13.77
Location				
Asia	9	125.27 ± 14.60	124.25 ± 14.20	126.30 ± 14.97
Non-Asia	2	128.44 ± 11.25	127.24 ± 11.80	130.40 ± 10.28
Duration				
< 10 weeks	6	128.66 ± 13.70	125.52 ± 14.35	126.99 ± 14.10
≥ 10 weeks	5	124.44 ± 14.15	123.99 ± 13.20	124.89 ± 15.14
NMN dose				
< 300 mg/d	5	128.66 ± 13.70	126.99 ± 14.10	125.52 ± 14.35
≥ 300 mg/d	6	124.44 ± 14.15	124.89 ± 15.14	123.99 ± 13.20
Baseline DBP (mmHg)				
Overall	11	77.10 ± 10.19	76.82 ± 10.66	77.40 ± 9.67
Age				
< 60 years	7	76.96 ± 10.14	76.40 ± 11.05	77.52 ± 9.14
≥ 60 years	4	77.33 ± 10.30	77.45 ± 10.09	77.19 ± 10.64
Baseline BMI				
< 25 kg/m^2	8	76.74 ± 11.23	76.19 ± 11.75	77.30 ± 10.70
≥ 25 kg/m^2	3	77.81 ± 7.71	77.96 ± 8.31	77.63 ± 6.97
Location				
Asia	9	77.19 ± 10.66	76.71 ± 11.21	77.67 ± 10.0
Non-Asia	2	76.64 ± 7.18	77.32 ± 7.96	75.53 ± 5.72
Duration				
< 10 weeks	6	78.57 ± 9.02	78.66 ± 9.64	78.47 ± 8.32
≥ 10 weeks	5	75.50 ± 11.13	74.73 ± 11.41	76.29 ± 10.84
NMN dose				
< 300 mg/d	5	78.57 ± 9.02	78.47 ± 8.32	78.66 ± 9.64
≥ 300 mg/d	6	75.50 ± 11.13	76.29 ± 10.46	74.73 ± 11.41

Figure S1 Sensitivity analysis of systolic blood pressure (A) and diastolic blood pressure (B).

Nakajima 2025a: NMN intake 750 mg/d; Nakajima 2025b: NMN intake 1500 mg/d.

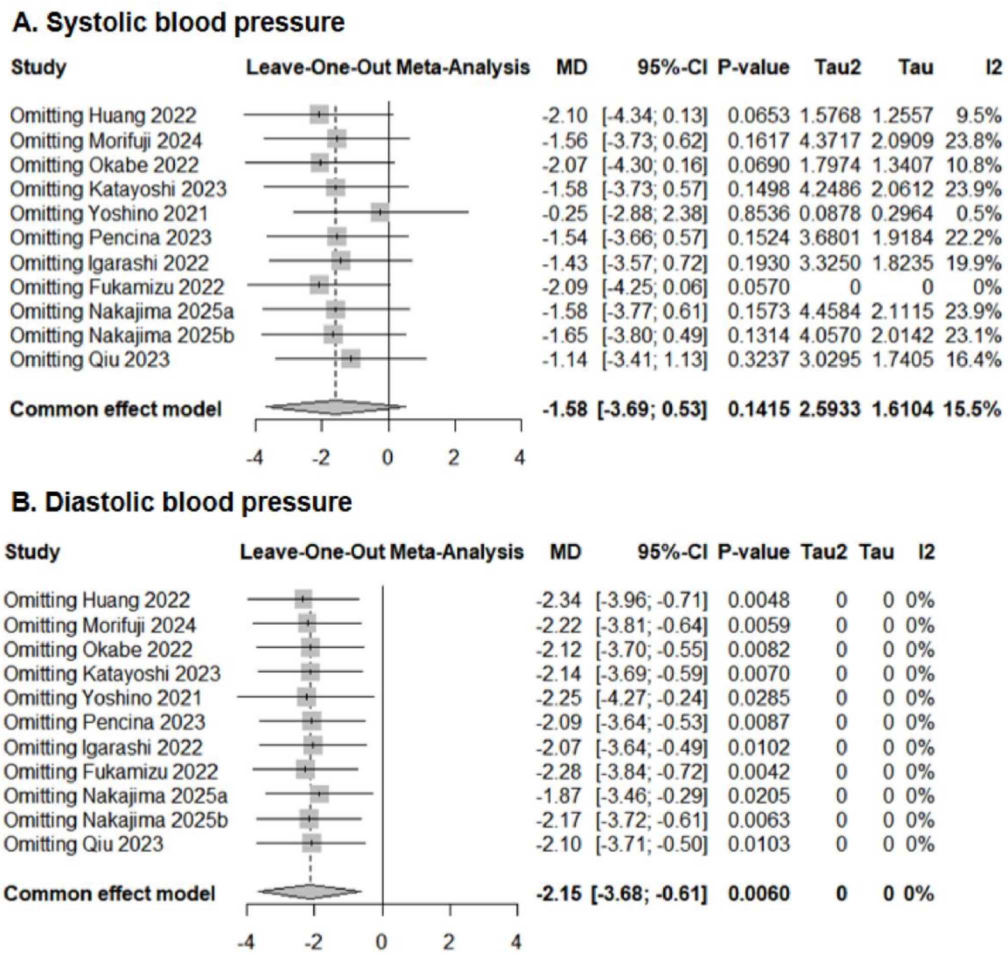

Figure S2 Forest plot of the effect of NMN supplementation on systolic blood pressure (A) and diastolic blood pressure (B) after excluding studies with high risk of bias. Nakajima 2025a: NMN intake 750 mg/d; Nakajima 2025b: NMN intake 1500 mg/d.

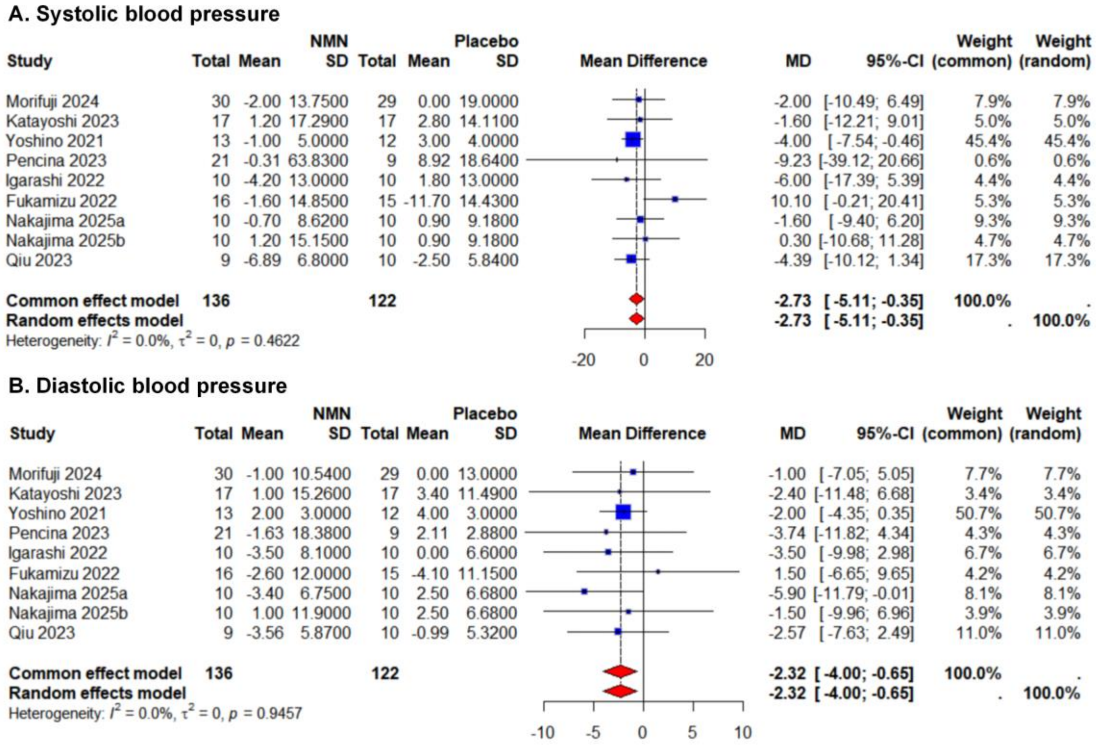

Supplement: Supplementary file 1 [file nutrients-18-00890-s001.zip › Supplementary Materials.pdf]
